# Pain Neuroscience Education for Children with Functional Abdominal Pain Disorders: A Randomized Comparative Pilot Study

**DOI:** 10.3390/jcm9061797

**Published:** 2020-06-09

**Authors:** Roselien Pas, Emma Rheel, Sophie Van Oosterwijck, Anthe Foubert, Robby De Pauw, Laurence Leysen, Ann Roete, Jo Nijs, Mira Meeus, Kelly Ickmans

**Affiliations:** 1 Pain in Motion Research Group (PAIN), Department of Physiotherapy, Human Physiology and Anatomy, Faculty of Physical Education and Physiotherapy, Vrije Universiteit Brussel, Laarbeeklaan 103, 1090 Brussels, Belgium; emma.rheel@vub.be (E.R.); laurence.leysen@vub.be (L.L.); jo.nijs@vub.be (J.N.); mira.meeus@uantwerpen.be (M.M.); kelly.ickmans@vub.be (K.I.); 2 Department of Physiotherapy, Human Physiology and Anatomy (KIMA), Faculty of Physical Education & Physiotherapy, Vrije Universiteit Brussel (VUB), Laarbeeklaan 103, 1090 Brussels, Belgium; 3 Department of Rehabilitation Sciences and Physiotherapy, Faculty of Medicine and Health Sciences, University of Antwerp (UA), D.S.022, 2610 Wilrijk, Belgium; Anthe.Foubert@uantwerpen.be; 4 Department of Experimental-Clinical and Health Psychology, Ghent University, Henri Dunantlaan 2, 9000 Ghent, Belgium; 5 Pain in Motion International Research Group; Sophie.VanOosterwijck@UGent.be; 6 Department of Rehabilitation Sciences, Faculty of Medicine and Health Sciences, Ghent University, Campus UZ, Corneel Heymanslaan 10, 9000 Ghent, Belgium; Robby.DePauw@UGent.be; 7 Research Foundation-Flanders (FWO), 1000 Brussels, Belgium; 8 Antwerp University Hospital, department of Pediatrics, 2610 Wilrijk, Belgium; ann.roete@uza.be; 9 Department of Physical Medicine and Physiotherapy, University Hospital Brussels, 1090 Brussels, Belgium

**Keywords:** hypnotherapy, catastrophizing, disability, fear, hyperalgesia

## Abstract

This article explores the effectiveness of a newly developed Pain Neuroscience Education program for children (PNE4Kids) with functional abdominal pain disorder (FAPD). Children (6–12 years) with FAPD were randomly assigned to 1) the experimental group (*n* = 14), participating in one hypnotherapy session (i.e., usual care) and one additional PNE4Kids session, or 2) the control group (*n* = 14), participating in two hypnotherapy sessions. Parental pain catastrophizing, the child’s functional disability (parental-proxy), pain-related fear (parent-proxy) and pain intensity, were assessed at baseline and one and three weeks after each therapy session. Pressure algometry and a conditioned pain modulation paradigm were performed at baseline and three weeks after completion of the last therapy session. Parents from both the experimental as well as the control group showed significantly less parental pain catastrophizing (*p* < 0.01). Children showed significantly less functional disability (*p* < 0.05), pain-related fear (*p* < 0.01) and local pressure pain sensitivity (*p* < 0.05) at short-term follow-up (three weeks after last intervention) in both groups. No significant (*p* > 0.05) between-group differences were found. Hypnotherapy combined with PNE4Kids did not result in better clinical outcomes compared to hypnotherapy alone. Study limitations include the application of one single PNE4Kids session and the short follow-up time.

## 1. Introduction

Functional Abdominal Pain Disorders (FAPDs) are prevalent in up to 13.5% of children and adolescents and have a significant impact on their health-related quality of life as well as their physical, academic and social activities and exposes children at a higher risk to the development of chronic pain and depressive symptoms later in life [1,2,3,4]. Children with FAPD might develop negative cognitions when they do not understand the origin of the pain they experience and should therefore be educated about their condition [5,6]. Current pain management in adolescents and in children with persistent pain often includes an educational component [7]. Nevertheless, this psychoeducation is focused on psychological aspects related to pain and on pain management strategies rather than on an explanation of the pain science, including underlying biological mechanisms of pain [8]. Indeed, it might be counterintuitive to patients (and parents) to advise them to adopt certain behaviors (i.e., resuming physical, social, and school activities) without decreasing the threat value of pain first.

Pain Neuroscience Education (PNE) therefore (1) includes a thorough explanation of pain science; (2) aims to reassure patients (and parents) by decreasing the threat value of pain; and (3) includes the integral role of psychosocial and physical factors in precipitating and maintaining pain. Therefore, PNE may prepare and prime children with chronic pain and their parents for biopsychosocial treatments [7,9,10,11]. PNE has been used in various adult chronic pain populations and has been shown to be effective in changing pain beliefs (i.e., pain catastrophizing) and pain coping strategies, improving disability, pain intensity, and health status [12,13].

The limited studies investigating PNE(-based) interventions in the pediatric chronic pain context, only include (healthy) adolescents and their parents [10,14,15]. Drawing upon available evidence in adult and adolescent samples, it is expected that PNE provided to children (6–12 years) and their parents will lead to improved pain-related outcomes in both the child and the parents. Involving the parents of children with FAPD in PNE sessions will facilitate an increased understanding of biopsychosocial factors that influence pain and might indirectly improve the child’s pain-related outcomes. Parents who catastrophize about their child’s pain show an encouragement of child pain behaviors [16], and promote more child anxiety and pain-related fear [17]. In addition, parents’ catastrophizing about their child’s pain has been found to predict child- and parent-reported functional disability and school attendance [18]. By including parents in therapy, they will learn how they can support their child in managing their symptoms.

To date, one of the most commonly used treatment modalities in children with FAPD is hypnotherapy. In general, hypnotherapy consists of exercises to induce general relaxation, as well as exercises to control abdominal pain and gut functions, all of which are aimed at increasing the child’s well-being. Hypnotherapy was proven to be effective in reducing pain in the short term in children with FAPD [19]. Given the innovative feature of PNE4Kids [7,20], ethical considerations have led us to use it in combination with the most commonly used therapy in Belgium, rather than investigating PNE4Kids as a stand-alone treatment. The present study examined whether children with FAPD and their parents receiving PNE4Kids in addition to standard care (i.e., hypnotherapy) would help improve parental pain catastrophizing and child’s functional disability (parental proxy) when compared to a group receiving only standard care. Secondly, we hypothesize that children with FAPD and their parents will show an improvement in pain intensity, pain-related fear, primary and secondary pressure hyperalgesia, and endogenous pain inhibition.

## 2. Experimental Section

### 2.1. Setting and Participants

Ethical approval for this 2-armed randomized controlled pilot study was obtained from the Ethics Committee of the University of Antwerp/Antwerp University Hospital and was part of another study examining endogenous pain modulation in children with FAPD [21]. The full study, including this part, was registered at clinicaltrials.gov (NCT02880332) in August 2016. Participants included boys and girls aged between 6 and 12 years who were presented at the pediatric department of the Antwerp University Hospital for abdominal pain. Other inclusion criteria were (1) the child had a history of pain for at least 3 months; (2) a diagnosis with FAPD by the pediatric abdominal pain specialist, based on the Rome III criteria [22]; and (3) the child was able to speak and read Dutch fluently (in an age-dependent manner). Exclusion criteria included children who (1) previously participated in one or more PNE session(s); (2) were diagnosed with a concomitant chronic disease (e.g., diabetes mellitus); (3) were born prematurely [23]; (4) previously participated in a study assessing experimental pain; or (5) presented with contra-indications to complete the experimental pain measures [24].

At the beginning of the study, children from the age of 12 and their parents were asked to sign the informed consent form. Participants were asked to refrain from performing heavy physical activity and from the intake of analgesics on the day of the pain testing procedure.

### 2.2. Study Flow

**Figure 1:**
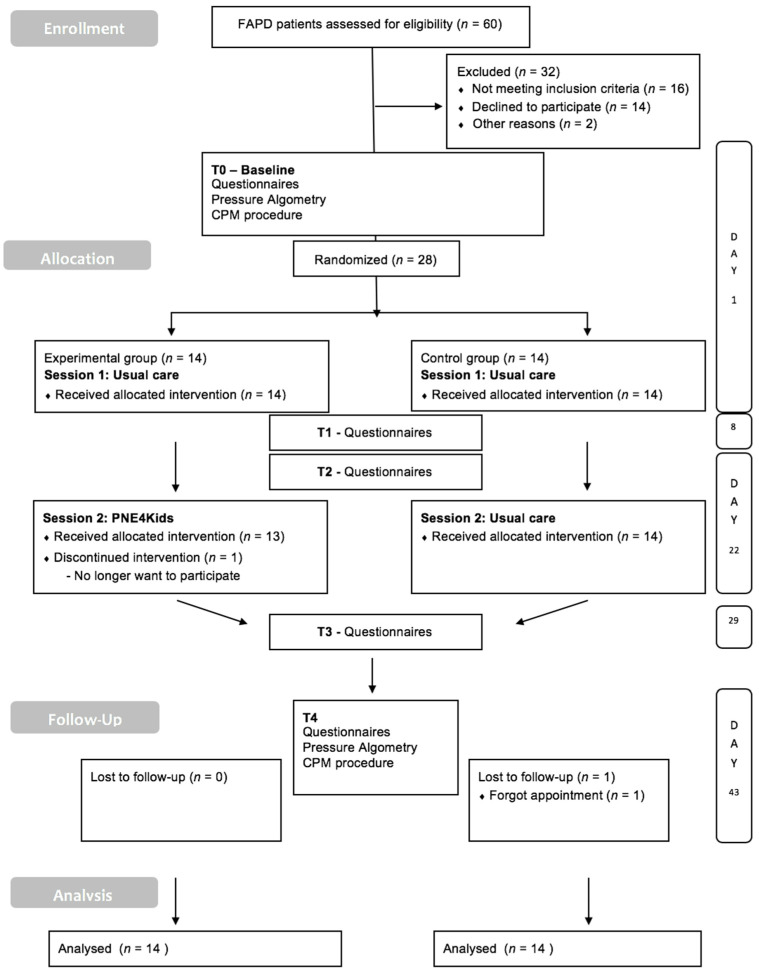
CONSORT Flow diagram.

Participants were randomized by (R.P.) to either the control or the experimental group by using a computerized random number generator for concealment. Both assessors (A.F. and S.V.O) were blind after the assignment. The procedure of the study and the CONSORT diagram including participant flow are presented in Figure 1.

### 2.3. Questionnaires

Parent: The Dutch Parent version of the Pain Catastrophizing Scale (PCS-P) was assessed, describing the different thoughts and feelings that parents may experience about their child’s pain [18]. Total scores range from 0–52, with higher scores indicating more pain catastrophizing about their child’s pain. In addition, three subscales can be calculated for (1) rumination; (2) magnification; and (3) helplessness. This scale has been reported to be a valid measurement tool [18]. The Dutch version of the Functional Disability Inventory-parent proxy (FDI-P) was used to measure difficulties in physical and psychosocial functioning during the past two weeks [25]. Total scores ranged from 0 to 60, with higher scores indicating greater functional disability. This tool has shown good validity and reliability measures in children with chronic abdominal pain [25]. The Dutch Fear of Pain Questionnaire-parent report (FOPQ-P) is a parent proxy report to assess a child’s pain-related fear and avoidance behavior. Total scores range from 0–92, with a higher score representing more fear [26]. This questionnaire has shown good psychometric properties [26].

Children: The Faces Pain Scale-Revised (FPS-R) was used to assess the child’s average abdominal pain intensity from the previous week. This scale consists of six faces, presented horizontally, which relate to a numeric value from 0 (“no pain”) to 10 (“the worst imaginable pain”). This scale has been recommended for research purposes based on its utility and psychometric features in children between 4 and 12 years [27,28]; it has also been used in cases of chronic pain [29].

### 2.4. Experimental Pain Measurements

The procedure of experimental pain testing has been described in detail previously [21]. Briefly, children completed two laboratory pain tasks in absence of their parents (pressure algometry and conditioned pain modulation by using the cold pressor task; more information is available in Appendix A).

### 2.5. Interventions

Control group: Children in the control group received two sessions of hypnotherapy (usual care). The first +/− 1 h interactive session was used to have the participant become familiar with the therapist (a nurse with specific expertise in hypnotherapy) and hypnosis techniques. These techniques included visualizations of a normal working gut (e.g., using metaphors adapted to the child’s interests, such as a car running at a normal speed). No fixed hypnotic scripts were used, the hypnosis technique was modified based on the child’s interests and by using feedback from the child. The second session included of a revision of the content given in the first session and additional breathing and relaxation exercises. Parents attended each therapy session, however they did not interact.

Experimental group: Children within the experimental group participated in one hypnotherapy session (usual care) plus Pain Neuroscience Education for children (PNE4Kids). The first session had the same content as the first session of the control group (described above). The second session, however, was different and consisted of a +/− 1 h one-on-one interactive educational session about the neurophysiology of pain using a board game. The PNE4Kids program was developed based on the book “Explain pain” by Butler and Moseley and includes an explanation and reassurance on the cause of pain, a brief summary of relevant pain mechanisms, and the integral role of psychosocial and physical factors in precipitating and maintaining pain [30]. The “Explain pain” material, which was initially written for adults, was adapted to the cognitive comprehensible stage of children and to the specific pain population (i.e., FAPD). More detailed information on these adaptations and on the exact content of the PNE4kids program can be read elsewhere [11] and is available via http://www.paininmotion.be/PNE4Kids. Parents attended each therapy session, however they did not interact.

### 2.6. Statistical Analyses

All data were analyzed using IBM SPSS Statistics for Macintosh, Version 24.0 Armonk, NY, USA IBM Corp. Analysis was done by intention to treat. Demographic variables were compared between groups by a Chi-square test for categorical variables and a Mann–Whitney U test for continuous variables with the significance level set at 5%. Based on these analyses, none of the demographic variables showed significant between-group differences (*p* > 0.05). To assess between-group difference in response to treatment, a random-intercept linear mixed models analysis was applied. A likelihood ratio test was implemented to assess the significance of the included variables. The model included treatment (experimental or control intervention), time (5 measurement points; T0–T4), and treatment × time as fixed effects together with a random intercept for each participant. Only significant interaction effects (i.e., treatment × time) were kept in the model. Primary analysis was performed on the two primary outcome measures; PSC-P (total score) and the FDI-P. Bonferroni-corrected significance levels were set at alpha = 0.025 to correct for multiple comparison (family-wise error rate (FWER) < 0.05). Besides, the estimated marginal mean for each time point was calculated based on the final model. Effect sizes of the mean time differences were calculated as the Cohen’s d.

## 3. Results

Patient enrollment took place between February 2017 and October 2018. A total of 28 children with FAPD were included in the study and received either two sessions of hypnotherapy (usual care) (control intervention; *n* = 14) or either a combination of one session of hypnotherapy usual care and one session of PNE4Kids (experimental intervention; *n* = 14) (Figure 1). Both intervention groups lost one participant; one participant did no longer want to participate (T2), while another participant forgot his/her follow-up appointment (T4). The demographic and baseline characteristics of the participants are presented in Table 1.

### 3.1. Primary Outcomes

**Figure 2:**
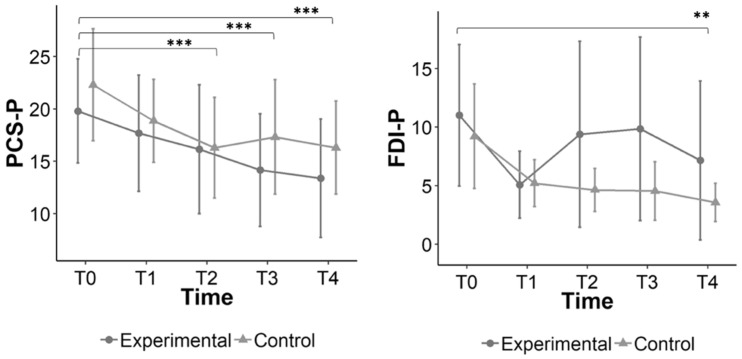
Graphs showing the mean difference over time for the primary outcomes with significant main time effect, *: *p* < 0.05; **: *p* < 0.01; ***: *p* < 0.00. Abbreviations: PCS-P: Pain Catastrophizing Scale for Parents (score: 0–52); FDI-P: Functional Disability Inventory-Parent proxy (score: 0–60);

The PCS-P and FDI-P did not show a significant interaction effect, although a significant main effect of time for both primary outcome measurements were found (Table 2, Figure 2).

For the total PCS-P significant improvements (medium-to-high effect sizes) from baseline were found at three weeks after session one (T2) (estimated marginal mean difference (diff.), 4.74; 95% CI, −0.31 to 6.03), one week after therapy completion (T3) (estimated marginal mean diff., 5.37, 95% CI, 2.16 to 8.51), and three weeks after therapy completion (T4) (estimated marginal mean diff., 6.07, 95% CI, 2.93 to 9.21).

For the FDI-P, significant improvements (medium effect size) were found from baseline to three weeks after therapy completion (T4) (estimated marginal mean diff., 5.05; 95% CI, 0.86 to 9.24).

The FOPQ-P showed improvements (medium effect size) from baseline to one week after therapy completion (T3) (estimated marginal mean diff., 5.52; 95% CI, 0.75 to 10.30) and three weeks after therapy completion (T4) (estimated marginal mean diff., 5.73; 95% CI, 1.02 to 10.45).

Table 3 shows the mean and standard error for the interaction effects of the primary outcome measures.

### 3.2. Secondary Outcomes

#### 3.2.1. Questionnaires

The PCS-P subscale rumination showed significant improvements (medium-to-high effect sizes) from baseline to three weeks after session one (T2) (estimated marginal mean diff., 1.85; 95% CI, 0.38 to 3.32), one week after therapy completion (T3) (estimated marginal mean diff., 1.93, 95% CI, 0.44 to 3.41), and three weeks after therapy completion (T4) (estimated marginal mean diff., 2.26; 95% CI, 0.79 to 3.73). For the PCS-P helplessness subscale, significant improvements (medium-to-high effect sizes) were found from baseline to one week after session one (T2) (estimated marginal mean diff., 1.86; 95% CI, 0.22 to 3.49), three weeks after session one (T2) (estimated marginal mean diff., 2.32; 95% CI, 0.70 to 3.95), one week after therapy completion (T3) (estimated marginal mean diff., 2.74; 95% CI, 1.10 to 4.39), and three weeks after therapy completion (T4) (estimated marginal mean diff., 3.03; 95% CI, 1.40 to 4.65).

The FOPQ-P showed improvements (medium effect size) from baseline to one week after therapy completion (T3) (estimated marginal mean diff., 5.52; 95% CI, 0.75 to 10.30) and three weeks after therapy completion (T4) (estimated marginal mean diff., 5.73; 95% CI, 1.02 to 10.45).

The FPS-R did not show a significant interaction effect, nor a significant main effect of time.

#### 3.2.2. Experimental Pain Measurements

None of the experimental pain measurements (locale and remote pressure hyperalgesia + conditioned pain modulation) showed a significant interaction effect (Table 2, Figure 2). However, for the PPT at the umbilicus test site a significant improvement (small effect size) was found at three weeks after therapy completion (T4) (estimated marginal mean diff., −0.15; 95% CI, −0.29 to 0.02).

## 4. Discussion

This study is the first to examine the combined effect of hypnotherapy and PNE4Kids in children with FAPD on parental pain catastrophizing and the parental estimation of the child’s functional disability. This exploratory randomized controlled trial was designed to gather valuable information regarding the effectiveness of PNE4Kids, to steer further research and clinics.

One session of PNE4Kids combined with one session of hypnotherapy (usual care) did not result in significantly better outcomes when compared to the control group receiving two sessions of usual care (i.e., hypnotherapy). Still, both intervention groups appeared to improve in the short-term (T4; at three weeks following the last intervention) regarding parental pain catastrophizing and functional disability. A variety of reasons might explain why no significant differences between the experimental and control group were found.

Both hypnotherapy and PNE4Kids are hypothesized to improve pain through similar mechanisms. The aim of PNE4Kids is to help children with FAPD and their parents in their understanding of the pain onset and progress of the disorder. The reconceptualization of pain might induce changes in insight and thereby changes in pain cognitions [11]. Hypnotherapy on the other hand, may bring about cognitive changes throughout by directly influencing cognitions, which helps to improve symptoms, or through influencing pain and gut functioning, which also leads to a change in cognition [31]. Hence, both treatment interventions might have induced comparable results, making it hard to find significant differences between both treatment groups.

Secondly, the sequence of the PNE4kids delivery (i.e., after the first usual care session) might have reduced its effects. Given the hypothesis that the reconceptualization of pain via PNE increases treatment responses, PNE is preferably given prior to additional exercises or cognitive and behavioral interventions [32]. In this study however, one PNE4Kids session was provided in addition to one usual care session to ensure the therapist giving the first intervention session was blind. Similar to pain management in adults with chronic pain, PNE should be used to enhance a time-contingent, cognition-targeted approach in the performance of (physical) activities [33], which are often impaired in children with FAPD [34]. Evidence of the isolated effect of PNE is available after one-on-one sessions in adults with chronic pain [35,36,37]. Although, the dosage of PNE sessions highly vary between studies, from one single session (of a duration of 3 h) in the study of Moseley et al. (2004) to four sessions in the study of Werner et al. (2016) Therefore it might be important to conduct follow-up educational sessions including a recapitulation of previous information in children. In addition, it might be useful to investigate the influence of other PNE delivery methods, including group sessions or blended learning [38].

Third, previous studies providing evidence for the effect of PNE in healthy children [10] and the context of pediatric pain has mainly focused on attitudes and knowledge of pain physiology, instead of pain-related outcomes such as physical functioning, pain intensity and pain-related fear [14,15]. Despite its frequent use in the evaluation of functional disability in the context of functional abdominal pain [39,40], it should be noted that the FDI was initially developed to assess perceived difficulty in a variety of activities that are relevant to children and adolescents, rather than to assess limitations specific to a particular pediatric condition such as FAPDs [25]. When analyzing the descriptive statistics of the separate items of the FDI, the two most reported disabling items were getting asleep and staying asleep (item 25; 89%) and eating regular meals (item 5; 64%). On the contrary, watching television (item 11) and staying awake all day (item 6) had the lowest ratings by parent report (respectively 7% and 18%). Including other outcomes, such as children’s knowledge of pain physiology, might have been valuable in assessing the additional use of PNE4Kids. To date, however, no valid and reliable assessment tool exists to assess children’s (6–12 years old) knowledge of pain.

There are some limitations to report regarding this study. First, we did not include a statistical analysis plan in the registered protocol of this study. Secondly, the use of parent-proxy questionnaires might have led to an under-estimation of children’s pain-related functioning, making it more difficult to detect treatment effects. Indeed, several items of the FDI refer to academic or social activities that parents may not directly observe. Including children from the age of six hampered the possibility of using direct measurements, since no questionnaire assessing pain catastrophizing was validated for our targeted age group. Third, the therapist providing the usual care was involved in both treatment arms, and although care was taken to ascertain that the PNE therapist was not involved in the usual care, it cannot be excluded that the therapist providing the usual care did not include aspects of PNE in their treatment. Study strengths include the randomized controlled design using balanced treatment arms, well-defined experimental treatment, a priori training of the therapists, clinical trial registration, and the use of blinded assessments.

## 5. Conclusions

Within this study, no significant differences were found between hypnotherapy (i.e., usual care including biomedical education) and hypnotherapy with PNE4Kids. Since additional PNE4Kids is not found to be worse either, further studies might examine treatment satisfaction and cost effectiveness among other features. Future studies should also investigate whether a combined approach of PNE4Kids applied as a preparatory therapy modality followed by a cognition-targeted active approach is able to improve pain-related outcomes in children with FAPD.

## Figures and Tables

**Table 1:** Demographic and Baseline Characteristics of the participants.

**Demographic**	**Participants No. (%)**	
Child sex		
Experimental group		
Female	9 (64.3)	
Male	5 (35.7)	
Control group		
Female	9 (64.3)	
Male	5 (35.7)	
Parental sex		
Experimental group		
Female	11 (78.6)	
Male	3 (21.4)	
Control group		
Female	11 (78.6)	
Male	3 (21.4)	
Parental history of chronic Pain		
Experimental group		
Yes	5 (35.7)	
No	9 (64.3)	
Control group		
Yes	4 (28.6)	
No	10 (71.7)	
Race child		
Experimental group		
Caucasian	13 (92.9)	
Non-Caucasian	1 (7.1)	
Control group		
Caucasian	13 (92.9)	
Non-Caucasian	1 (7.1)	
Parental educational level		
Experimental group		
Low (≤high school level education)	5 (35.7)	
Middle (Bachelor’s degree or equivalent)	6 (42.9)	
High (≥Master’s degree)	3 (21.4)	
Control group		
Low (≤high school level education)	5 (35.7)	
Middle (Bachelor’s degree or equivalent)	5 (35.7)	
High (≥Master’s degree)	4 (28.6)	
**Demographic**	**Mean (SD)**	**Median (IQR) (Range)**
Child age, years		
Experimental group	9.21 (1.53)	9.00 (3.00) (7.00–12.00)
Control group	8.71 (1.73)	9.00 (3.25) (6.00–11.00)
Parental age, years		
Experimental group	39.57 (4.18)	39.00 (5.25) (35.00–49.00)
Control group	37.57 (4.65)	38.00 (7.75) (29.00–44.00)
Pain duration, months		
Experimental group	19.71 (12.68)	17.00 (15.75) (4.00–48.00)
Control group	28.36 (21.40)	24.00 (39.25) (3.00–70.00)
Pain episodes, n/month		
Experimental group	21.29 (8.73)	23.50 (14.25) (3.00–31.00)
Control group	20.64 (14.73)	16.50 (17.75) (3.00–60.00)
**Baseline**	**Mean (SD)**	**Median (IQR) [Range]**
FDI-P score		
Experimental group	11.00 (11.60)	6.50 (16.25) (0.00–43.00)
Control group	9.21 (8.54)	7.00 (6.25) (2.00–34.00)
FPS-R score		
Experimental group	3.71 (2.20)	4.00 (2.50) (0.00–8.00)
Control group	5.14 (2.80)	5.00 (4.50) (0.00–10.00)
FOPQ-P score		
Experimental group	41.50 (14.85)	37.50 (23.75) (24.00–73.00)
Control group	31.71 (9.23)	34 (14.25) (12.00–42.00)
PCS-P score		
Experimental group	19.79 (9.54)	19.00 (12.25) (3.00–41.00)
Control group	22.29 (10.25)	23.50 (9.50) (2.00–40.00)
PPT Trapezius, kg/cm²		
Experimental group	1.41 (0.85)	1.17 (0.61) (0.73–4.00)
Control group	1.15 (0.43)	1.02 (0.70) (0.68–2.05)
PPT Umbilicus, kg/cm²		
Experimental group	1.04 (0.52)	1.05 (0.77) (0.13–2.10)
Control group	0.95 (0.32)	0.84 (0.36) (0.50–1.61)
PPT Tibia, kg/cm²		
Experimental group	2.61 (1.12)	2.59 (1.44) (1.19–5.00)
Control group	2.47 (1.05)	2.36 (1.39) (1.08–4.50)
CPM, absolute difference		
Experimental group	−0.01 (0.59)	−0.07 (0.51) (−0.63–1.76)
Control group	−0.28 (0.37)	−0.19 (1.10) (−1.08–0.30)
CPM, % change		
Experimental group	−7.90 (26.45)	−6.75 (40.17) (−50.00–44.12)
Control group	−32.64 (39.88)	−22.54 (73.06) (−105.19–24.49)

Abbreviations: IQR: Interquartile range; SD: Standard deviation; PCS-P: Pain Catastrophizing Scale for Parents (score: 0–52); FDI-P: Functional Disability Inventory-Parent proxy (score: 0–60); FOPQ-P: Fear of Pain Questionnaire-Parent version (score: 0–92); FPS-R: Faces Pain Scale-Revised (range 0–10); PPT: Pain Pressure Threshold; CPM: Conditioned Pain Modulation; CPT: Cold Pressor Task; Calculations: CPM absolute difference = (PPT Trapezius–PPT Trapezius during CPM); CPM Percentage change = (((PPT Trapezius-PPT Trapezius during CPM)/PPT Trapezius during CPM)*100).

**Table 2:** Main group, time, and interaction effect for the primary and secondary outcomes.

Model	Group			Time		Group × Time	
	AIC	F (df)	*P*-Value	F (df)	*P*-Value	F (df)	*P*-Value
Primary outcomes							
PCS-P	825.5	0.292 (1)	0.593	9.831 (4)	<0.001	NA	
FDI-P	882.1	0.959 (1)	0.337	3.272 (4)	0.014	NA	
Secondary outcomes							
POFQ-P	928.0	1.620 (1)	0.214	4.527 (4)	0.002	NA	
FPS-R	628.2	2.622 (1)	0.117	2.536 (4)	0.045	NA	
PCS-P (Rum.)	626.0	0.069 (1)	0.791	6.800 (4)	<0.001	NA	
PCS-P (Magn.)	463.8	0.445 (1)	0.511	2.440 (4)	0.052	NA	
PCS-P (Help.)	650.3	0.439 (1)	0.514	8.947 (4)	<0.001	NA	
PPT Trapezius	92.8	1.356 (1)	0.255	0.074 (1)	0.788	NA	
PPT Umbilicus	54.7	0.207 (1)	0.653	5.745 (1)	0.024	NA	
PPT Tibia	146.0	0.524 (1)	0.476	4.226 (1)	0.050	NA	
CPM, Abs Diff.	72.788	1.928 (1)	0.177	3.176 (1)	0.087	NA	
CPM, % change	492.291	3.343 (1)	0.080	1.063 (1)	0.314	NA	

Abbreviations: PCS-P: Pain Catastrophizing Scale for Parents (score: 0–52); FDI-P: Functional Disability Inventory-Parent proxy (score: 0–60); FOPQ-P: Fear of Pain Questionnaire-Parent version (score: 0–92); FPS-R: Faces Pain Scale-Revised (range 0–10); PPT: Pain Pressure Threshold; CPM: Conditioned Pain Modulation; CPT: Cold Pressor Task; AIC: Akaike information criterion; (df): Degrees of freedom; NA: Not applicable; Calculations: CPM absolute difference = (PPT Trapezius–PPT Trapezius during CPM); CPM Percentage change = (((PPT Trapezius-PPT Trapezius during CPM)/PPT Trapezius during CPM)*100).

**Table 3:** Mean and standard error for the interaction effects of the primary outcome measures.

Primary Outcomes and Their Measurement in Time (T)	Experimental Group Mean (S.E.)	Control Group Mean (S.E)
PCS-P		
0	19.786 (2.554)	22.286 (2.554)
1	17.541 (2.602)	18.857 (2.554)
2	16.385 (2.580)	16.286 (2.554)
3	14.385 (2.580)	17.021 (2.576)
4	13.616 (2.580)	16.286 (2.554)
FDI-P		
0	11.000 (2.593)	9.214 (2.593)
1	7.540 (2.676)	5.214 (2.593)
2	8.806 (2.637)	4.643 (2.593)
3	9.268 (2.637)	4.751 (2.631)
4	6.576 (2.637)	3.571 (2.593)

Abbreviations: PCS-P: Pain Catastrophizing Scale for Parents (score: 0–52); FDI-P: Functional Disability Inventory-Parent proxy (score: 0–60); S.E: Standard Error.

## Appendix A

Pressure hyperalgesia was assessed with the Force Dial model FDK 40 algometer (Wagner Instruments, Greenwich, UK) by administering pressure via a small flat rubber disc with a surface area of 1 cm to the umbilical (local site) [41], and the trapezial [41] and tibial regions (both remote sites) [42] at the dominant side. The children were instructed to say “stop” at the moment when the sensation of the mechanical stimulus applied to their body changed from pressure to pain. A total of three measurements (pressure pain threshold (PPT) measurements) were performed at each testing site, with 30 s between each test. The mean of the last two PPTs per test site was calculated and used for further analyses.

To assess efficiency of endogenous pain inhibition, the conditioned pain modulation (CPM) paradigm was performed by combining pressure algometry and the cold pressor task. The water circulator apparatus cooled water to 12 °C ± 1 °C and had a continuous flow and circulation rate. The child was instructed to immerse its non-dominant hand in the circulating water. Following an initial immersion of 20 s, the PPT at the trapezial region was reassessed three times, with an inter-stimulus duration of 30 s. Immediately after the last PPT measurement, the child was instructed to withdraw its hand out of the water and was offered a towel to dry and warm the hand. Again, the mean of the latter two PPTs was calculated. The outcome measure for CPM was obtained by the following formula: ((PPT preCPM-PPT postCPM)/PPT pre-CPM)*100.

## Appendix B

As this paper was somewhat exploratory in nature, we deviated slightly from our registered protocol, and we report those deviations here in the spirit of transparency.

We originally planned to explore the influence of the intervention on the primary outcome parental pain catastrophizing (PCS-P), but decided to add a second primary outcome measure focusing on the child, this being a pain-related disability. Based on the results of a recent Cochrane literature review which included 18 randomized controlled trials, it became clear that studies investigating pain-related disability following hypnotherapy in children with functional abdominal pain were lacking [43].

We planned to report on the child’s pain related to fear using parent-reported (The Dutch Fear of Pain Questionnaire–parent report) [26] as well as child-reported measurements (The Kuttner Anxiety Scale). The reason why we did not report the results on the child-reported Kuttner Anxiety Scale was mainly because we could not find any other study reporting the reliability and validity measures of this scale.
